# Waiting Time and Focus of Physical Therapy for Children with Cerebral Palsy in Saudi Arabia: Parents’ Report

**DOI:** 10.3390/children12050544

**Published:** 2025-04-24

**Authors:** Abdulrhman Mashabi, Maysoun N. Saleh, Ahmad A. Alharbi, Abdulaziz A. Albalwi, Hani F. Albalawi, Qais Al-Bakri

**Affiliations:** 1 Department of Physical Therapy, College of Medical Rehabilitation Sciences, Taibah University, Al-Madinah Al-Munawarah 42353, Saudi Arabia; 2 Department of Health Rehabilitation Sciences, Faculty of Applied Medical Sciences, University of Tabuk, Tabuk 71491, Saudi Arabia; m-saleh@ut.edu.sa (M.N.S.); aaalharbi@ut.edu.sa (A.A.A.); aa-albalwi@ut.edu.sa (A.A.A.); hf_albalawi@ut.edu.sa (H.F.A.); 3 Department of Physiotherapy, School of Rehabilitation Sciences, The University of Jordan, Amman 11942, Jordan; 4 Department of General Surgery, School of Medicine, The University of Jordan, Amman 11942, Jordan

**Keywords:** cerebral palsy (CP), children, Saudi Arabia, service utilization, physical therapy, gross motor function classification system (GMFCS)

## Abstract

**Background:** Physical therapy is crucial in the rehabilitation of children with cerebral palsy (CP), aiming to enhance motor function, postural control, and functional independence. **Objective:** The study explored the current physical therapy interventions for children with CP in Saudi Arabia, including waiting time, the most used interventions, the focus of therapy, and parents’ desired goals. **Methods:** A cross-sectional study was conducted involving 215 children with CP (aged 6 months to 18.2 years). Face-to-face surveys were conducted to collect data on CP classification (based on the Gross Motor Function Classification System), age at first referral, types of interventions used, intervention goals, and parents’ desired goals for their children. **Results:** Children with severe CP (non-ambulators) received physical therapy services significantly earlier than those with milder involvement (ambulators). The most commonly used interventions were therapeutic exercises and home exercises, followed by standing frames. Hydrotherapy was the least utilized intervention. The focus of therapy was mainly on joints and muscles, as well as mobility and transfers. **Conclusions:** The study underscores the need to identify and refer children with CP for physical therapy. The findings suggest further investigation into barriers to utilizing certain interventions like hydrotherapy and emphasize the need for more inclusive goal-setting processes in the rehabilitation of children with CP based on both physical therapy and parent perspectives.

## 1. Introduction

Cerebral palsy (CP) is a prevalent neurological disorder caused by non-progressive disturbances to the developing brain [1]. Common neuropathological findings in cerebral palsy, such as periventricular leukomalacia, cortical and subcortical lesions, hypoxic–ischemic encephalopathy, and brain malformations, are often linked to prenatal, perinatal, or early postnatal factors [2,3,4].

The prevalence of CP varies significantly across countries due to differences in healthcare systems, neonatal care quality, and population-specific risk factors [2]. In high-income countries (HICs) such as Australia, Sweden, and the United States, recent estimates show a declining trend in CP prevalence, with current rates ranging from 1.4 to 1.6 per 1000 live births, largely attributed to improvements in perinatal care and neonatal intensive care unit (NICU) management. In contrast, low- and middle-income countries (LMICs) such as India and Uganda report higher prevalence rates, often exceeding 3.0 per 1000 live births, driven by limited access to quality maternal and neonatal healthcare [5,6]. In Saudi Arabia, the reported prevalence of CP is approximately 2.34 per 1000 live births, which is higher than the average for HICs and the global estimate of 2.11 per 1000 live births [7,8]. This relatively elevated rate may be influenced by factors such as a high rate of consanguineous marriages, regional disparities in neonatal care, and a lack of comprehensive national surveillance systems [7]. When compared to other Arabic-speaking countries, where the pooled prevalence is around 1.8 per 1000 live births, Saudi Arabia still shows a higher burden, underscoring the need for enhanced early intervention services and national-level registries to monitor and address CP more effectively [7].

CP can lead to a variety of motor impairments and functional limitations, including muscle stiffness (spasticity), poor coordination, difficulty with balance and posture, involuntary movements, and challenges with fine motor skills [1]. These impairments, in turn, impact functional independence and reduce the quality of life.

Physical therapy plays a crucial role in the rehabilitation of children with CP [9]. Physical therapy involves a wide range of interventions aimed at improving muscle strength and flexibility, postural control, and coordination. In addition, physical therapy programs aim to help individuals with CP reach their maximum physical potential, enhance their functional activity and participation, leading to improvement in overall quality of life [9,10,11]. However, parental satisfaction with the care provided by physical therapists can influence the effectiveness of physical therapy interventions [12,13]. Parental perceptions of their child’s condition, needs, and expected outcomes significantly shape their attitudes and behaviors toward healthcare management [12,13,14,15,16]. Therefore, considering parental perspectives of the rehabilitation processes is vital for improving outcomes and tailoring interventions to meet their needs.

Studies showed that when physical therapy goals align with the priorities and expectations of parents, children are more likely to experience improved engagement in therapy, better adherence to home programs, and enhanced overall outcomes [12,13,14,15,16,17,18,19,20,21,22]. In addition, by including parents’ input, physical therapists can develop more personalized rehabilitation plans that address the physical, emotional, and social needs of both the child and the family. Therefore, researchers recommended the use of a family-centered approach to CP rehabilitation in order to incorporate parents’ goals in the rehabilitation program [12,13,14,15,16,17,18,19,20,21,22].

The focus of physical therapy interventions may be described using the International Classification of Functioning, Disability, and Health (ICF) framework (ICF; World Health Organization, 2001) [23]. ICF provides a common language among healthcare professionals to describe goals in rehabilitation. For example, physical therapy interventions may be directed at impairments (primary or secondary), activity limitations, and/or participation restrictions. Based on this framework, several studies have explored therapy goals that parents prioritize for their children’s rehabilitation [24,25,26]. For example, Jindal et al. [24] indicated that Indian parents were more concerned about treating body structure and function impairments and achieving independent walking than Canadian parents, who focused more on activity at school and home and participation in accessible communities. Another study by Nguyen et al. [25] explored the parents’ goals of therapy based on ICF. In this study, all participating parents reported body structure and function goals, primarily focusing on reducing muscle tone and improving range of motion in the extremities. Over half of the parents identified goals related to activity, whereas fewer than 5% reported goals focused on participation [25].

In Saudi Arabia, the extent to which parents perceive physical therapy interventions as family-centered was explored by Alharbi and Albalwi [27]. However, to date, there is no study that has explored the actual focus of physical therapy services offered to children with CP in Saudi Arabia and the parents’ desired goals of physical therapy for their children. Therefore, this study aimed to explore physical therapy management for children with CP in Saudi Arabia. Our specific aims included the following: (1) exploring the child’s age at which he/she received the first physical therapy session, (2) the current physical therapy practices and techniques and how do they differ by region and child’s level of impairment, (3) the focus of current physical therapy interventions (based on the ICF domains), (4) parents’ desired focus of physical therapy for their children, and (5) differences between the current focus of physical therapy interventions and parents’ desired focus for their children were also explored.

## 2. Materials and Methods

### 2.1. Study Design and Ethical Considerations

This cross-sectional explorational study was part of a nationwide investigation conducted in Saudi Arabia from February to August 2022, aiming to explore the characteristics of children with cerebral palsy and the services they received [28]. The study protocol followed the ethical criteria outlined by the National Committee of Bioethics (NCBE), in accordance with the existing norms and guidelines. Ethical approval for the study was granted by the local ethics commission of the University of Tabuk (UT-175-39-2022).

### 2.2. Participants

A convenience sample of children with CP and their parents was recruited from 14 cities across 8 of the 13 provinces in Saudi Arabia. Parents were invited to participate during their children’s physical therapy sessions at rehabilitation centers or hospitals. Research assistants (RAs), who were physical therapists, explained the study’s objectives and procedures and obtained signed informed consent from parents if approved to participate. Eligible participants were parents or legal guardians and their children, who had a confirmed diagnosis of CP by a pediatric neurologist and who were receiving physical therapy services. The RAs, who were not the children’s primary physical therapists to reduce potential social desirability or courtesy bias, conducted face-to-face interviews and documented responses using a standardized child and family information form. To ensure consistency in data collection methods, the RAs underwent a two-hour training workshop conducted by the research team to review measurements, methods, and address any queries. This wide range of recruitment sites was intended to enhance the generalizability of the findings and capture variations in clinical practice.

### 2.3. Measures

#### 2.3.1. The Gross Motor Function Classification System—Expanded and Revised (GMFCS)

The gross motor function level of the child was determined using the GMFCS. RAs, who are pediatric physical therapists and who had received specialized training on the use of GMFCS, determined the best level of the GMFCS that described the child’s current abilities and limitations in gross motor function. The GMFCS is a valid and reliable classification system of a child’s gross motor function. It comprises five distinct levels and is designed for children diagnosed with CP. The GMFCS classifies children’s gross motor function, with the focus on their functional mobility skills and limitations. Children classified as Level I exhibit minor limitations in their gross motor function, while children at Level V have maximal limitations and are dependent in their mobility. The interrater reliability coefficient (kappa) was 0.55 for children under the age of 2, and 0.75 for those aged 2 to 12 [29,30].

#### 2.3.2. Family and Child Information Form

A self-report questionnaire was constructed to collect information from families of children with CP. Information collected included demographics such as the child’s age and sex, in addition to family and household data, such as parents’ educational level, household income, and geographic region of domicile. Parents also reported on their child’s primary and secondary impairments—visual impairments, hearing impairments, speech disorders, nutritional deficiencies, learning disabilities, attention deficits, epilepsy, respiratory conditions, musculoskeletal disorders, obesity, and pain problems [28].

#### 2.3.3. Physical Therapy Services Information

Parents reported on their children’s age when he/she had the first referral for physical therapy, and the child’s age when he/she had the first physical therapy session. To determine the most frequent physical therapy techniques and interventions used during the past 12-month period, parents were given a list including the standing frame, therapeutic exercise, play therapy (such as the use of toys, ball, or games to engage children in treatment), hydrotherapy, assistive device, and home exercise and were asked to rank them according to the frequency of use, with number 1 as the most frequent intervention used and 6 as the least frequently use. The choices included all ICF domains (body function and structure, activity, participation, and environment).

In addition, parents were asked about the focus (goal) of the physical therapy intervention provided to their children. They were given choices including joint and muscle exercises (stretching and strengthening), functional activities, eating and dressing, playing, and communication and social integration. They were asked to rate them from 1 (the main focus) to 5 (the least focus). Finally, parents were asked about their perspective on desired physical therapy goals for their children and to rank them in terms of priority from 1 (the most important to them) to 5 (the least important to them).

### 2.4. Data Analyses

Data analysis was performed using SPSS version 25 (SPSS Inc., Chicago, IL, USA). Children were grouped into two groups based on the GMFCS: ambulatory group (levels I to III—ambulatory with or without limitations) and non-ambulatory group (levels IV to V—non-ambulatory). The eight provinces of residence were grouped into five geographic regions: north, south, central, east, and west (Table 1). Descriptive statistics were utilized to describe sample characteristics, including frequencies (and proportions) for categorical data and means and standard deviations for continuous data. The independent samples t-tests were used to compare group differences if the data were normally distributed; otherwise, the Mann–Whitney U test was applied for non-normally distributed data. Choen’s Kappa coefficient was used to examine the agreement between the actual physical therapy’s focus/goals and parents’ desired goals. The Chi square test was used to examine the variability of treatment techniques by region of domicile and by the GMFCS groups. The alpha level was 0.05 for all analyses.

## 3. Result

### 3.1. Participants’ Characteristics

The study included 215 children diagnosed with CP, ranging in age from 6 months to 18.2 years, with a mean age of 6.2 ± 3.7 years, and their parents. Table 1 displays the characteristics of the participating children. The survey respondents consisted of 81.6% mothers, 16.5% fathers, 0.9% both parents, and 1% individuals identified as others (e.g., aunts).

**Time until receiving the first physical therapy session:**

There was an approximate 10-month difference in the timing of the first referral to physical therapy between ambulatory and non-ambulatory groups. The ambulatory group had the first referral at an average age of 26.10 ± 29.11 months, while the non-ambulatory group was referred earlier, at 16.89 ± 14.72 months, with a statistically significant difference (*p* = 0.002). Similarly, the mean age at which children received their first physical therapy session was significantly later in the ambulatory group (26.80 ± 28.91 months) compared to the non-ambulatory group (18.82 ± 16.0 months), with a *p*-value of 0.009.

### 3.2. Most Frequently Used Physical Therapy Interventions for Children with CP in Saudi Arabia

**Figure 1:**
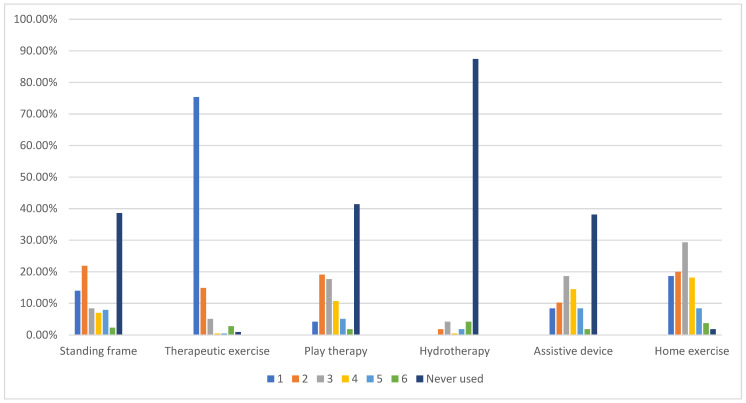
Parents’ report on the use of physical therapy interventions for their children with CP in Saudi Arabia. CP, cerebral palsy; (rank 1), most frequently used; (rank 6), less frequently used.

Figure 1 presents parents’ reports on the use of various physical therapy interventions for their children with CP in Saudi Arabia. Therapeutic exercises were identified as the most frequently used intervention, with 75.35% of children receiving this therapy and only 0.92% never having received it. Additionally, home exercise programs were also commonly practiced, with 18.60% of parents reporting it as one, the first most frequently used intervention, and 20% ranked it as two, the second most frequently used. The use of standing frames showed considerable variability. For example, 21.86% of parents ranked it as two, the second most frequently used, whereas 38.60% indicated that their children had never used a standing frame in physical therapy sessions. Furthermore, hydrotherapy was the least utilized intervention, with 87.44% of parents reporting that their children had never participated in this therapy. Similarly, play therapy was not widely adopted, with 41.40% of parents reporting that it was never used in their children’s treatment. However, 19.07% and 17.7% of parents gave it ranks two and three, respectively.

### 3.3. Parent Reports on the Focus (Goal) of Physical Therapy Interventions Provided to Their Children with CP in Saudi Arabia Based on the ICF Model

**Figure 2:**
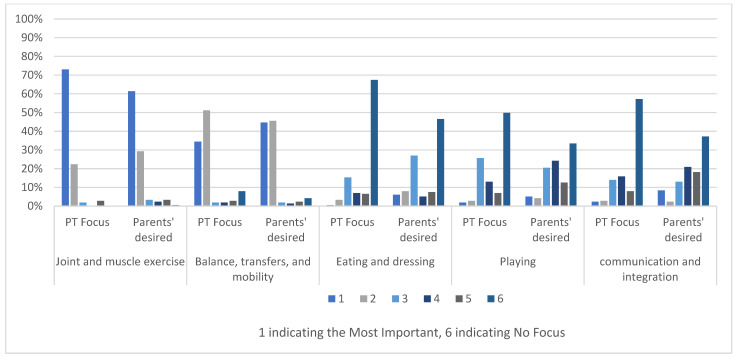
Parents’ report on physical therapy focuses and their desired focus for children with CP in Saudi Arabia. CP, cerebral palsy; (rank 1), highest focus; (rank 5), lowest focus; 6, no focus.

Figure 2 highlights the current focus of physical therapy as reported by parents and parents’ desired focus for their children with CP, based on the ICF model. A total of 73.02% of parents reported that their children’s actual physical therapy focused on ‘Joint and muscle exercises’ and gave it rank one, which indicates the major focus of therapy. in addition, 61.39% of parents ranked joint and muscle exercises as the number one desired focus. The second area of focus was ‘balance, transfers and mobility’, and it was also the second most desired focus by parents.

There was moderate to fair agreement between actual and desired focus, highest for joint and muscle exercises (Cohen’s Kappa coefficient 0.484) and lowest for eating and dressing (0.357), Table 2.

Cohen suggested the Kappa result be interpreted as follows: values ≤ 0 as indicating no agreement and 0.01–0.20 as none to slight, 0.21–0.40 as fair, 0.41–0.60 as moderate, 0.61–0.80 as substantial, and 0.81–1.00 as almost perfect agreement [22].

### 3.4. Variability of Physical Therapy Techniques Used by Geographic Region and by the GMFCS Groups

Table 3 shows the distribution of physical therapy treatment techniques used by the geographic region and by the GMFCS groups. The use of play therapy, hydrotherapy, and assistive devices significantly differed by region. In addition, the use of play and assistive devices differed by the GMFCS groups.

## 4. Discussion

### 4.1. Time to Receive the First Session of Physical Therapy for Children with CP

This is the first study that has sought to explore the physical therapy services for children with CP in Saudi Arabia based on the parents’ report. Non-ambulatory children (GMFCS IV and V) had received statistically significantly earlier physical therapy services significantly earlier than ambulatory children (GMFCS I, II, and III) by approximately 10 months. Receiving a diagnosis and intervention of CP has been reported as challenging in the medical system in many countries due to fewer healthcare providers, lack of time, essential knowledge, appropriate skills, and adequate resources to effectively identify and refer a child with special needs at an early stage [31]. It is likely that in Saudi Arabia, children with more severe motor impairments are diagnosed and referred for therapy sooner than those with less severe impairments. Novak et al. [31] and Shevell et al. [32] found that children with more severe motor impairments (GMFCS level IV or V) are typically identified and referred for physical therapy earlier than those with milder forms of cerebral palsy, as the functional limitations are more apparent early in life. Further research is necessary to explore how to promote early identification of children at risk for CP and thus provide earlier physical therapy interventions for them in Saudi Arabia.

### 4.2. Physical Therapy Interventions for Children with CP in Saudi Arabia

Therapeutic exercises followed by home exercises were identified by parents as the most frequent therapies used for their children. There is strong evidence of effectiveness for the use of home exercises for children with CP [31,33]. However, research did not support the use of therapeutic exercises [31,33]. These findings on the effectiveness of these two interventions should encourage physical therapists to enhance the use of home programs for children with CP, but need not discourage the use of therapeutic exercises. We recommend more research to explore what type of therapeutic exercises are being used for children with CP in Saudi Arabia (e.g., stretching, strengthening, aerobics…, etc.). We also recommend more research to explore which of these exercises are more beneficial for these children.

Hydrotherapy was the least-used intervention with our participants. The literature has shown conflicting evidence on the effectiveness of hydrotherapy for children with CP [34,35,36,37]. In addition, there are several barriers to its use for children with CP, including the availability of appropriate facilities, trained personnel, transportation, financial limitations, and a lack of awareness among healthcare providers regarding the benefits of aquatic therapy [35]. Additionally, concerns about the safety and suitability of hydrotherapy for children with severe motor impairments further limit its accessibility. Given these barriers, further research is needed to better understand the obstacles to implementing hydrotherapy as a rehabilitation intervention for children with CP in Saudi Arabia and to further explore the effectiveness of its use for children with CP.

Similarly, the use of a standing frame was not very common in our cohort. Evidence on the use of assistive devices, in general, for the management of children with CP is also controversial [34,35,36,37]. In addition, there is also conflicting evidence on the use of standing frames for children with CP [38,39,40]. Goodwin et al. [38] explored the views of stakeholders, including healthcare professionals and parents, regarding the clinical benefits and challenges of using standing frames. Participants reported various clinical indications and perceived benefits, including improvements in bladder and bowel function, motor abilities, and peer interaction. They also noted additional benefits, such as improved bone mineral density and the prevention of hip dislocation. Identified factors influencing the prescription of standing frames included funding limitations, environmental constraints, personal preferences, and challenges faced by the child and family. However, this study explored stakeholders’ opinions on the benefits of standing frames, without actually measuring these benefits. Therefore, in Saudi Arabia, more research is needed to explore the benefits of the use of standing frames for children with CP, explore barriers to their use, and also investigate parents’ and physical therapists’ opinions on the usefulness of their use for children with CP.

### 4.3. Parent Perspective on the Physical Therapy Goal of Children with CP in Saudi Arabia Based on the ICF Model

This study highlights both the agreement and differences between the focus of physical therapy and parents’ preferences as the most important aspect to focus the therapy on for their children with CP. There was moderate to fair agreement between the actual physical therapy focus and parents’ desires. Actual and desired focus had close agreement on the priority of interventions focusing on joint and muscle exercises as well as balance, transfers, and mobility. In addition, there was a lower priority for activities such as eating, dressing, and playing. This was also true for communication and integration.

Demont et al. [33] have developed evidence-based, implementable motor rehabilitation guidelines for children with CP. They reported strong recommendations for gait training and physical activities for all children and adolescents with CP. Our data showed that physical therapy focus was on balance, transfers (sitting, standing), and mobility, which were shown to be best practices [33]. These findings are encouraging, and we recommend exploring the best knowledge transfer approaches to update physical therapists about best practices based on research evidence.

In their recommendations for interventions to improve physical function for children with CP, Jackman et al. [41] have recommended setting client-chosen goals and that interventions should target the child’s chosen goals. Therefore, we highly stress the importance of effective communication and exchange of information between parents and therapists. This will ensure agreement of both sides on therapy priority and focus for the child. A recent study on the provision of family-centered care for children with CP in Saudi Arabia revealed that services are less family-centered, where families expressed the need for more information about their children and services [27]. Indeed, more exchange of information will enable parents to make informed choices about their children’s therapy. Physical therapists are also encouraged to seek evidence-based practice in light of recent research and practice guidelines. However, as suggested by Wiart et al. [42], other factors may affect the choice of therapy goals and focus, such as therapists’ experiences and beliefs, and, therefore, clinical decision making cannot always be based on ‘best research evidence’. Therefore, open communication channels between physical therapists and parents will ensure agreement on therapy goals.

The parents indicated that eating and dressing, play, and communication and integration were not priorities in their children’s actual physical therapy. In addition, our participant parents did not think that these areas were desired priorities for their children’s physical therapy goals. One possible explanation may be that these areas are more addressed by occupational therapy and speech therapy, not physical therapy. A study by Saleh et al. [28] found that most of the children with CP in Saudi Arabia did not receive occupational therapy and speech therapy services, which may explain part of the reason why these areas are not receiving enough attention. However, further investigation is needed to explore rehabilitation services directed to activities of daily living, play, communication, and integration for children with CP in Saudi Arabia.

Overall, the findings from this study offer valuable insights into current physical therapy practices and goal-setting priorities for managing CP from the parents’ perspective. Future research should aim to explore factors that lead to these preferences.

### 4.4. Limitations

This study used parents’ reports on their children’s health and services received. Therefore, further research is needed to explore actual practices and goals of therapy as reported by the physical therapists working with children with CP. In addition, although our sample was large enough and was collected from many sites across the country, generalization of the results to all children with CP in Saudi Arabia should be done with caution. Some areas were represented with more participants than other areas [28]. Further investigation is warranted to include more participants and validate the results.

## 5. Conclusions

This study provides valuable insights into physical therapy services for children with CP in Saudi Arabia from a parental perspective. The delay in referral of the less severely involved children to physical therapy calls for more efforts to identify children at risk for CP and make sure they receive physical therapy services as early as possible. In addition, more exchange of information between physical therapists and parents is expected to increase agreement on therapy goals and focus. The decreased actual and desired focus on functional activities and participation emphasizes the necessity for further studies to explore factors that led to these preferences in providing physical therapy for children with CP in Saudi Arabia. Addressing these insights will facilitate a more comprehensive approach to managing CP.

## Figures and Tables

**Table 1:** Participating parents and children’s characteristics, **N = 215**.

Characteristic	n (%)	Characteristic	n (%)
**Household Region**		**Sex**	
South	51 (23.72%)	Male	126 (58.60%)
Center	49 (22.79%)	Female	89 (41.40%)
East	23 (10.70%)	**GMFCS Level**	
West	85 (39.53%)	I–III (Group 1)	117 (55.42%)
North	7 (3.26%)	IV–V (Group 2)	98 (45.58%)
**Respondent Relationship**		**Age Group (years)**	
Mother	174 (80.9%)	0–2	53 (24.7%)
Father	33 (15.3%)	3–5	70 (32.6%)
Both Parents	2 (0.9%)	6–11	80 (37.2%)
Others	6 (2.8%)	12–18	12 (5.6%)
Comorbidities *			
Premature birth (<37 weeks)	96 (44.7%)	Eye problems (strabismus, lenses)	16 (7.4%)
Visual impairment	26 (12.1%)	Hearing impairment	6 (2.8%)
Delayed speech	48 (22.3%)	Cognitive/intellectual disability	51 (23.7%)
Seizures	14 (6.5%)	Asthma	5 (2.3%)
Learning difficulties	30 (14.0%)	Septal defect	4 (1.9%)
Feeding tube	11 (5.1%)	Ventriculoperitoneal (VP) shunt	3 (1.4%)
**Type of CP**			
Spastic-Diplegia	74 (34.4%)	Spastic-Hemiplegia	22 (10.2%)
Spastic-Quadriplegia	63 (29.3%)	Spastic-Monoplegia	5 (2.3%)
Spastic-Triplegia	10 (4.7%)	Hypotonia	16 (7.4%)
Dyskinetic	6 (2.8%)	Ataxic	5 (2.3%)
Mixed	7 (3.3%)	Unknown	7 (3.3%)
**Physiotherapy Frequency**		**Physiotherapy Session Duration**	
<54 sessions (<1/week)	45 (21.0%)	Range: 10–120 min, Mean:	47.4 min/session
54–108 sessions (1–2/week)	125 (58.1%)		
>108 sessions (>2/week)	45 (20.9%)		

* The parent can choose more than one comorbidity.

**Table 2:** Cohen’s kappa for the agreement between physical therapy focuses and parents’ desired focus for children with CP in Saudi Arabia (N:215).

Task	Joint and Muscle Exercise (Body Function)	Functional Activities (Activities)	Eating and Dressing (Activities and Participation)	Playing (Activities and Participation)	Communication and Participation (Activities and Participation)
Cohen’s Kappa coefficient	0.484	0.391	0.357	0.478	0.437

**Table 3:** Use of PT techniques by region and GMFCS groups.

PT Technique		Standing Frame N (%)		Therapeutic Exercises N (%)		Play Therapy *^,†^ N (%)		Hydrotherapy * N (%)		Assistive Devices *^,†^ N (%)		Home Exercises N (%)	
**Region**		No	Yes	No	Yes	No	Yes	No	Yes	No	Yes	No	Yes
	South	19 (37.3)	32 (62.2)	0 (0)	51 (100)	30 (58.8)	21 (41.2)	51 (100)	0 (0)	39 (76.5)	12 (23.5)	0 (0)	51 (100)
	Center	21 (42.9)	28 (57.1)	2 (4.1)	47 (95.9)	18 (36.7)	31 (63.3)	46 (93.9)	3 (6.1)	15 (30.6)	34 (69.4)	0 (0)	49 (100)
	East	8 (34.8)	15 (65.2)	0 (0)	23 (100)	10 (43.5)	13 (56.5)	10 (43.5)	13 (56.5)	3 (13)	20 (87)	0 (0)	23 (100)
	West	34 (40)	51 (60)	0 (0)	85 (100)	30 (35.3)	55 (64.7)	74 (87.1)	11 (12.9)	25 (29.4)	60 (70.6)	4 (4.7)	81 (95.3)
	North	1 (14.3)	6 (85.7)	0 (0)	7 (100)	1 (14.3)	6 (85.7)	7 (100)	0 (0)	0 (0)	7 (100)	0 (0)	7 (100)
**GMFCS**													
	Group 1	48 (41)	69 (59)	1 (0.9)	116 (99.1)	40 (34.2)	77 (65.8)	98 (83.8)	19 (16.2)	37 (31.6)	80 (68.4)	3 (2.6)	114 (97.4)
	Group 2	35 (35.7)	63 (64.3)	1 (1)	97 (99)	49 (50)	49 (50)	90 (91.8)	8 (8.2)	45 (45.9)	53 (54.1)	1 (1)	97 (99)

* Significant correlation with region, *p* ≤ 0.05. ^†^ Significant correlation with GMFCS *p* ≤ 0.05.

## Data Availability

Data supporting the findings of this study are available from the corresponding author upon reasonable request. The data are not publicly available due to ethical and patient privacy considerations.
